# [Bis(diphenyl­phosphan­yl)dimethyl­silane-κ^2^ *P*,*P*′]tetra­carbonyl­chromium(0)

**DOI:** 10.1107/S1600536810043679

**Published:** 2010-10-31

**Authors:** Normen Peulecke, Stephan Peitz, Bernd H. Müller, Anke Spannenberg, Uwe Rosenthal

**Affiliations:** a Leibniz-Institut für Katalyse e. V. an der Universität Rostock, Albert-Einstein-Strasse 29a, 18059 Rostock, Germany

## Abstract

The title compound, [Cr(C_26_H_26_P_2_Si)(CO)_4_], was obtained by the reaction of Ph_2_PSiMe_2_PPh_2_ with Cr(CO)_6_ in refluxing toluene by ligand exchange. The CrC_4_P_2_ coordination geometry at the Cr atom is distorted octa­hedral, with a P—Cr—P bite angle of 80.27 (1)°.

## Related literature

For the synthesis of Ph_2_PSiMe_2_PPh_2_, see: Hassler & Seidl (1988 ▶). The mol­ecular and crystal structures of the tetra­carbonyl tungsten complex of [(^*i*^Pr_2_N)_2_BP(H)]_2_SiMe_2_ and the tetra­carbonyl molybdenum complex of (PhPHSiMe_2_)_2_ were presented by Chen *et al.* (1999 ▶) and Sheldrick & Borkenstein (1977 ▶), respectively.
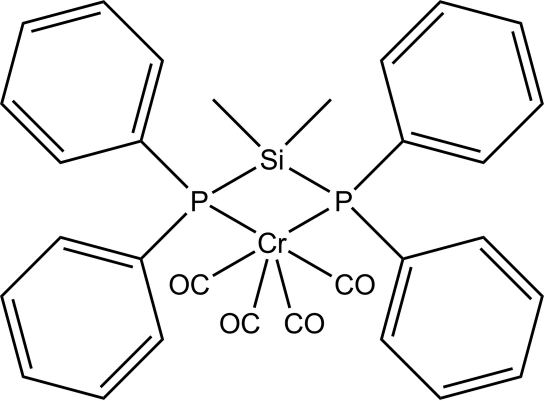


## Experimental

### 

#### Crystal data

[Cr(C_26_H_26_P_2_Si)(CO)_4_]
*M* *_r_* = 592.54
Monoclinic, 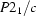

*a* = 13.7832 (4) Å
*b* = 11.9204 (2) Å
*c* = 18.1329 (5) Å
β = 102.073 (2)°
*V* = 2913.36 (13) Å^3^
*Z* = 4
Mo *K*α radiation
μ = 0.58 mm^−1^
*T* = 200 K
0.45 × 0.40 × 0.38 mm

#### Data collection

Stoe IPDS II diffractometer
Absorption correction: numerical (*X-SHAPE* and *X-RED32*; Stoe & Cie, 2005 ▶) *T*_min_ = 0.773, *T*_max_ = 0.867
47168 measured reflections
6688 independent reflections
5370 reflections with *I* > 2σ(*I*)
*R*_int_ = 0.032

#### Refinement

*R*[*F* ^2^ > 2σ(*F* ^2^)] = 0.026
*wR*(*F* ^2^) = 0.065
*S* = 0.93
6688 reflections
345 parameters
6 restraints
H-atom parameters constrained
Δρ_max_ = 0.32 e Å^−3^
Δρ_min_ = −0.33 e Å^−3^

### 

Data collection: *X-AREA* (Stoe & Cie, 2005 ▶); cell refinement: *X-AREA*; data reduction: *X-AREA*; program(s) used to solve structure: *SHELXS97* (Sheldrick, 2008 ▶); program(s) used to refine structure: *SHELXL97* (Sheldrick, 2008 ▶); molecular graphics: *XP* in *SHELXTL* (Sheldrick, 2008 ▶); software used to prepare material for publication: *SHELXL97*.

## Supplementary Material

Crystal structure: contains datablocks I, global. DOI: 10.1107/S1600536810043679/cv2781sup1.cif

Structure factors: contains datablocks I. DOI: 10.1107/S1600536810043679/cv2781Isup2.hkl

Additional supplementary materials:  crystallographic information; 3D view; checkCIF report

## Figures and Tables

**Fig. 1.**
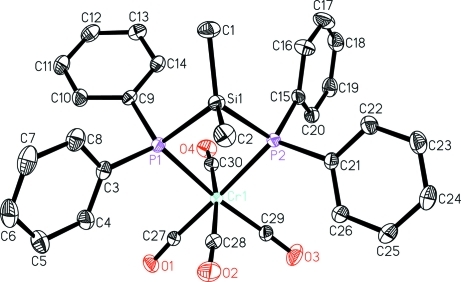
The molecular structure of the title compound showing the atom-labelling scheme. H atoms are omitted for clarity. Displacement ellipsoids are drawn at the 30% probability level.

## supplementary crystallographic information

### Comment

Disphosphines are widely used as chelate ligands for complex formation. Among them the silicon-bridged species are not common. There are only few examples of structurally characterized complexes with a SiMe_2_ bridge (Chen *et al.*, 1999) or SiMe_2_SiMe_2_ bridge (Sheldrick & Borkenstein, 1977). In the present publication, we report on the formation and molecular structure of the title compound, which was observed to be the single product of a complex formation of Ph_2_PSiMe_2_PPh_2_ with Cr(CO)_6_. The synthesis of the starting ligand was already reported by Hassler & Seidl (1988).

In the molecular structure of the title complex the chelating disphosphine and four carbonyl ligands are coordinated to the Cr atom (Fig. 1). The coordination geometry at the metal center is best described as distorted octahedral. The observed bite angle P—Cr—P is 80.27 (1)° and the P—Si—P angle of the complexed ligand is 85.31 (2)°.

### Experimental

Cr(CO)_6_ (175 mg, 0.8 mmol) was added to a solution of Ph_2_PSiMe_2_PPh_2_ (321 mg, 0.75 mmol) in 20 ml of toluene and the resulting solution was stirred at reflux temperature for 72 h. Subsequently, the formed yellow solution was cooled down to 0°C and filtered. Toluene was removed in vacuum and the product was extracted with dichloromethane. The major part of dichloromethane was removed and the remaining solution was over-layered with *n*-hexane to get single crystals of the title compound. The yellow compound was fully characterized by standard analytical methods *e.g.*^31^P NMR: (CD_2_Cl_2_): 5.9 p.p.m.

### Refinement

All H atoms were placed in idealized positions with d(C—H) = 0.98 (CH_3_) and 0.95 Å (CH) and refined using a riding model with *U*_iso_(H) fixed at 1.5 *U*_eq_(C) for CH_3_ and 1.2 *U*_eq_(C) for CH.

### Crystal data

**Table:**

[Cr(C_26_H_26_P_2_Si)(CO)_4_]	*F*(000) = 1224
*M**_r_* = 592.54	*D*_x_ = 1.351 Mg m^−^^3^
Monoclinic, *P*2_1_/*c*	Mo *K*α radiation, λ = 0.71073 Å
Hall symbol: -P 2ybc	Cell parameters from 13232 reflections
*a* = 13.7832 (4) Å	θ = 2.0–29.6°
*b* = 11.9204 (2) Å	µ = 0.58 mm^−^^1^
*c* = 18.1329 (5) Å	*T* = 200 K
β = 102.073 (2)°	Prism, yellow
*V* = 2913.36 (13) Å^3^	0.45 × 0.40 × 0.38 mm
*Z* = 4	

### Data collection

**Table:**

Stoe IPDS II diffractometer	6688 independent reflections
Radiation source: fine-focus sealed tube	5370 reflections with *I* > 2σ(*I*)
graphite	*R*_int_ = 0.032
ω scans	θ_max_ = 27.5°, θ_min_ = 2.1°
Absorption correction: numerical (*X-SHAPE* and *X-RED32*; Stoe & Cie, 2005)	*h* = −17→17
*T*_min_ = 0.773, *T*_max_ = 0.867	*k* = −15→15
47168 measured reflections	*l* = −23→23

### Refinement

**Table:**

Refinement on *F*^2^	Primary atom site location: structure-invariant direct methods
Least-squares matrix: full	Secondary atom site location: difference Fourier map
*R*[*F*^2^ > 2σ(*F*^2^)] = 0.026	Hydrogen site location: inferred from neighbouring sites
*wR*(*F*^2^) = 0.065	H-atom parameters constrained
*S* = 0.93	*w* = 1/[σ^2^(*F*_o_^2^) + (0.0437*P*)^2^] where *P* = (*F*_o_^2^ + 2*F*_c_^2^)/3
6688 reflections	(Δ/σ)_max_ = 0.001
345 parameters	Δρ_max_ = 0.32 e Å^−^^3^
6 restraints	Δρ_min_ = −0.33 e Å^−^^3^

### Special details

**Table:**

Geometry. All e.s.d.'s (except the e.s.d. in the dihedral angle between two l.s. planes) are estimated using the full covariance matrix. The cell e.s.d.'s are taken into account individually in the estimation of e.s.d.'s in distances, angles and torsion angles; correlations between e.s.d.'s in cell parameters are only used when they are defined by crystal symmetry. An approximate (isotropic) treatment of cell e.s.d.'s is used for estimating e.s.d.'s involving l.s. planes.
Refinement. Refinement of *F*^2^ against ALL reflections. The weighted *R*-factor *wR* and goodness of fit *S* are based on *F*^2^, conventional *R*-factors *R* are based on *F*, with *F* set to zero for negative *F*^2^. The threshold expression of *F*^2^ > σ(*F*^2^) is used only for calculating *R*-factors(gt) *etc*. and is not relevant to the choice of reflections for refinement. *R*-factors based on *F*^2^ are statistically about twice as large as those based on *F*, and *R*-factors based on ALL data will be even larger.

### Fractional atomic coordinates and isotropic or equivalent isotropic displacement parameters (Å^2^)

**Table:**

	*x*	*y*	*z*	*U*_iso_*/*U*_eq_	
C1	0.25499 (13)	0.34671 (15)	0.23882 (9)	0.0452 (4)	
H1A	0.2964	0.3105	0.2079	0.068*	
H1B	0.2941	0.4035	0.2711	0.068*	
H1C	0.1979	0.3826	0.2060	0.068*	
C2	0.13677 (12)	0.12988 (15)	0.23902 (10)	0.0445 (4)	
H2A	0.0795	0.1647	0.2057	0.067*	
H2B	0.1136	0.0746	0.2714	0.067*	
H2C	0.1782	0.0926	0.2086	0.067*	
C3	0.37225 (9)	0.03094 (13)	0.35878 (8)	0.0312 (3)	
C4	0.38402 (12)	−0.06394 (13)	0.40336 (10)	0.0411 (4)	
H4	0.3647	−0.0619	0.4507	0.049*	
C5	0.42314 (14)	−0.16177 (14)	0.38092 (10)	0.0525 (4)	
H5	0.4309	−0.2259	0.4127	0.063*	
C6	0.45082 (13)	−0.16610 (15)	0.31233 (9)	0.0535 (5)	
H6	0.4776	−0.2332	0.2965	0.064*	
C7	0.43945 (13)	−0.07262 (14)	0.26685 (10)	0.0553 (5)	
H7	0.4588	−0.0753	0.2196	0.066*	
C8	0.40018 (12)	0.02518 (15)	0.28928 (9)	0.0442 (4)	
H8	0.3922	0.0889	0.2571	0.053*	
C9	0.43399 (9)	0.25333 (12)	0.40684 (7)	0.0277 (3)	
C10	0.52916 (10)	0.20972 (14)	0.41518 (9)	0.0378 (3)	
H10	0.5384	0.1311	0.4113	0.045*	
C11	0.61087 (11)	0.28078 (16)	0.42918 (10)	0.0455 (4)	
H11	0.6758	0.2503	0.4358	0.055*	
C12	0.59842 (12)	0.39468 (16)	0.43350 (9)	0.0438 (4)	
H12	0.6546	0.4428	0.4432	0.053*	
C13	0.50415 (12)	0.43899 (14)	0.42375 (9)	0.0386 (3)	
H13	0.4952	0.5179	0.4251	0.046*	
C14	0.42244 (10)	0.36853 (13)	0.41193 (8)	0.0330 (3)	
H14	0.3579	0.3994	0.4073	0.040*	
C15	0.13366 (9)	0.43218 (12)	0.41500 (8)	0.0302 (3)	
C16	0.14302 (13)	0.51650 (14)	0.36418 (10)	0.0456 (4)	
H16	0.1494	0.4976	0.3145	0.055*	
C17	0.14317 (15)	0.62837 (15)	0.38556 (12)	0.0561 (5)	
H17	0.1496	0.6854	0.3503	0.067*	
C18	0.13416 (12)	0.65736 (14)	0.45713 (12)	0.0493 (4)	
H18	0.1344	0.7341	0.4714	0.059*	
C19	0.12477 (12)	0.57488 (15)	0.50772 (10)	0.0446 (4)	
H19	0.1183	0.5946	0.5572	0.053*	
C20	0.12470 (10)	0.46281 (13)	0.48722 (9)	0.0355 (3)	
H20	0.1185	0.4064	0.5229	0.043*	
C21	−0.00463 (9)	0.25993 (12)	0.35370 (8)	0.0291 (3)	
C22	−0.06017 (11)	0.33304 (14)	0.30152 (9)	0.0394 (3)	
H22	−0.0289	0.3955	0.2837	0.047*	
C23	−0.16100 (12)	0.31502 (16)	0.27540 (10)	0.0466 (4)	
H23	−0.1985	0.3656	0.2401	0.056*	
C24	−0.20685 (11)	0.22462 (16)	0.30030 (10)	0.0466 (4)	
H24	−0.2760	0.2130	0.2825	0.056*	
C25	−0.15301 (11)	0.15113 (16)	0.35085 (11)	0.0479 (4)	
H25	−0.1847	0.0880	0.3675	0.058*	
C26	−0.05175 (11)	0.16869 (14)	0.37797 (9)	0.0392 (3)	
H26	−0.0149	0.1177	0.4133	0.047*	
C27	0.29769 (10)	0.06277 (12)	0.55229 (8)	0.0303 (3)	
C28	0.16332 (10)	0.02616 (13)	0.43194 (9)	0.0353 (3)	
C29	0.12597 (10)	0.16240 (13)	0.54054 (8)	0.0340 (3)	
C30	0.29387 (10)	0.27186 (13)	0.53968 (8)	0.0318 (3)	
Cr1	0.221253 (14)	0.156127 (18)	0.482132 (12)	0.02433 (6)	
O1	0.34093 (8)	0.00645 (10)	0.59947 (6)	0.0440 (3)	
O2	0.13246 (10)	−0.05665 (11)	0.40470 (8)	0.0575 (3)	
O3	0.06848 (9)	0.16388 (12)	0.57808 (7)	0.0554 (3)	
O4	0.34046 (9)	0.33533 (10)	0.57916 (7)	0.0500 (3)	
P1	0.32570 (2)	0.16071 (3)	0.392418 (19)	0.02575 (8)	
P2	0.12811 (2)	0.28288 (3)	0.39145 (2)	0.02589 (8)	
Si1	0.21062 (3)	0.23959 (3)	0.29856 (2)	0.02921 (9)	

### Atomic displacement parameters (Å^2^)

**Table:**

	*U*^11^	*U*^22^	*U*^33^	*U*^12^	*U*^13^	*U*^23^
C1	0.0530 (9)	0.0510 (10)	0.0323 (8)	−0.0054 (8)	0.0102 (7)	0.0108 (7)
C2	0.0419 (8)	0.0442 (10)	0.0423 (9)	−0.0046 (7)	−0.0029 (7)	−0.0077 (7)
C3	0.0247 (6)	0.0372 (8)	0.0317 (7)	−0.0008 (5)	0.0059 (5)	−0.0067 (6)
C4	0.0453 (8)	0.0373 (9)	0.0439 (9)	0.0068 (7)	0.0163 (7)	−0.0026 (7)
C5	0.0559 (10)	0.0401 (10)	0.0636 (12)	0.0125 (8)	0.0175 (9)	−0.0059 (9)
C6	0.0445 (9)	0.0551 (12)	0.0605 (11)	0.0092 (8)	0.0098 (8)	−0.0249 (9)
C7	0.0479 (9)	0.0807 (14)	0.0400 (9)	0.0054 (9)	0.0151 (7)	−0.0227 (10)
C8	0.0421 (8)	0.0581 (11)	0.0341 (8)	0.0032 (7)	0.0118 (6)	−0.0048 (8)
C9	0.0248 (6)	0.0349 (8)	0.0238 (6)	−0.0035 (5)	0.0063 (5)	−0.0002 (5)
C10	0.0285 (7)	0.0390 (9)	0.0449 (9)	0.0006 (6)	0.0058 (6)	0.0002 (7)
C11	0.0256 (7)	0.0560 (11)	0.0543 (10)	−0.0031 (7)	0.0069 (7)	−0.0009 (8)
C12	0.0358 (8)	0.0536 (11)	0.0423 (9)	−0.0163 (7)	0.0091 (6)	−0.0058 (8)
C13	0.0454 (8)	0.0366 (9)	0.0355 (8)	−0.0089 (6)	0.0121 (6)	−0.0067 (6)
C14	0.0309 (7)	0.0380 (8)	0.0313 (7)	−0.0009 (6)	0.0089 (5)	−0.0038 (6)
C15	0.0233 (6)	0.0259 (7)	0.0396 (8)	0.0006 (5)	0.0024 (5)	0.0016 (6)
C16	0.0587 (10)	0.0330 (9)	0.0449 (9)	−0.0010 (7)	0.0102 (8)	0.0067 (7)
C17	0.0694 (12)	0.0301 (9)	0.0673 (13)	−0.0016 (8)	0.0110 (10)	0.0113 (8)
C18	0.0420 (9)	0.0285 (8)	0.0741 (13)	0.0022 (7)	0.0047 (8)	−0.0062 (8)
C19	0.0377 (8)	0.0407 (9)	0.0547 (10)	0.0033 (7)	0.0084 (7)	−0.0117 (8)
C20	0.0326 (7)	0.0326 (8)	0.0420 (8)	0.0011 (6)	0.0090 (6)	−0.0003 (6)
C21	0.0233 (6)	0.0309 (7)	0.0327 (7)	0.0015 (5)	0.0050 (5)	−0.0012 (6)
C22	0.0331 (7)	0.0380 (9)	0.0441 (9)	0.0013 (6)	0.0012 (6)	0.0060 (7)
C23	0.0339 (8)	0.0514 (11)	0.0486 (10)	0.0068 (7)	−0.0049 (7)	0.0029 (8)
C24	0.0256 (7)	0.0601 (11)	0.0510 (9)	−0.0017 (7)	0.0015 (6)	−0.0074 (8)
C25	0.0309 (7)	0.0517 (11)	0.0605 (11)	−0.0099 (7)	0.0080 (7)	0.0045 (9)
C26	0.0295 (7)	0.0384 (9)	0.0481 (9)	−0.0022 (6)	0.0044 (6)	0.0072 (7)
C27	0.0280 (6)	0.0306 (7)	0.0345 (7)	0.0031 (5)	0.0119 (5)	0.0025 (6)
C28	0.0300 (7)	0.0318 (8)	0.0452 (8)	−0.0014 (6)	0.0104 (6)	0.0039 (7)
C29	0.0270 (6)	0.0393 (8)	0.0356 (7)	0.0043 (6)	0.0064 (6)	0.0072 (6)
C30	0.0320 (7)	0.0318 (8)	0.0325 (7)	0.0022 (6)	0.0085 (6)	0.0040 (6)
Cr1	0.02209 (10)	0.02451 (11)	0.02716 (11)	0.00111 (8)	0.00691 (7)	0.00371 (9)
O1	0.0445 (6)	0.0457 (7)	0.0418 (6)	0.0156 (5)	0.0093 (5)	0.0138 (5)
O2	0.0552 (7)	0.0373 (7)	0.0805 (9)	−0.0142 (6)	0.0154 (7)	−0.0111 (6)
O3	0.0383 (6)	0.0841 (10)	0.0499 (7)	0.0068 (6)	0.0230 (5)	0.0083 (7)
O4	0.0540 (7)	0.0442 (7)	0.0476 (7)	−0.0103 (6)	0.0007 (5)	−0.0063 (6)
P1	0.02266 (15)	0.02917 (18)	0.02576 (16)	−0.00015 (13)	0.00579 (12)	0.00080 (14)
P2	0.02295 (15)	0.02462 (18)	0.02950 (17)	0.00002 (12)	0.00413 (12)	0.00330 (14)
Si1	0.02813 (18)	0.0322 (2)	0.02609 (18)	−0.00233 (15)	0.00286 (14)	0.00240 (15)

### Geometric parameters (Å, °)

**Table:**

C1—Si1	1.8588 (16)	C15—P2	1.8281 (15)
C1—H1A	0.9800	C16—C17	1.389 (3)
C1—H1B	0.9800	C16—H16	0.9500
C1—H1C	0.9800	C17—C18	1.373 (3)
C2—Si1	1.8580 (16)	C17—H17	0.9500
C2—H2A	0.9800	C18—C19	1.369 (3)
C2—H2B	0.9800	C18—H18	0.9500
C2—H2C	0.9800	C19—C20	1.387 (2)
C3—C4	1.380 (2)	C19—H19	0.9500
C3—C8	1.394 (2)	C20—H20	0.9500
C3—P1	1.8277 (15)	C21—C26	1.385 (2)
C4—C5	1.3813 (14)	C21—C22	1.392 (2)
C4—H4	0.9500	C21—P2	1.8350 (13)
C5—C6	1.3760 (15)	C22—C23	1.388 (2)
C5—H5	0.9500	C22—H22	0.9500
C6—C7	1.3756 (15)	C23—C24	1.373 (3)
C6—H6	0.9500	C23—H23	0.9500
C7—C8	1.3818 (15)	C24—C25	1.369 (3)
C7—H7	0.9500	C24—H24	0.9500
C8—H8	0.9500	C25—C26	1.395 (2)
C9—C14	1.388 (2)	C25—H25	0.9500
C9—C10	1.3896 (19)	C26—H26	0.9500
C9—P1	1.8308 (13)	C27—O1	1.1509 (17)
C10—C11	1.389 (2)	C27—Cr1	1.8455 (14)
C10—H10	0.9500	C28—O2	1.1452 (19)
C11—C12	1.373 (3)	C28—Cr1	1.8874 (16)
C11—H11	0.9500	C29—O3	1.1477 (18)
C12—C13	1.379 (2)	C29—Cr1	1.8530 (14)
C12—H12	0.9500	C30—O4	1.1424 (18)
C13—C14	1.385 (2)	C30—Cr1	1.8860 (15)
C13—H13	0.9500	Cr1—P1	2.3877 (4)
C14—H14	0.9500	Cr1—P2	2.3983 (4)
C15—C16	1.388 (2)	P1—Si1	2.2731 (5)
C15—C20	1.390 (2)	P2—Si1	2.2798 (5)
			
Si1—C1—H1A	109.5	C18—C19—H19	119.8
Si1—C1—H1B	109.5	C20—C19—H19	119.8
H1A—C1—H1B	109.5	C19—C20—C15	120.70 (15)
Si1—C1—H1C	109.5	C19—C20—H20	119.7
H1A—C1—H1C	109.5	C15—C20—H20	119.7
H1B—C1—H1C	109.5	C26—C21—C22	118.69 (13)
Si1—C2—H2A	109.5	C26—C21—P2	119.72 (11)
Si1—C2—H2B	109.5	C22—C21—P2	121.59 (11)
H2A—C2—H2B	109.5	C23—C22—C21	120.26 (15)
Si1—C2—H2C	109.5	C23—C22—H22	119.9
H2A—C2—H2C	109.5	C21—C22—H22	119.9
H2B—C2—H2C	109.5	C24—C23—C22	120.42 (15)
C4—C3—C8	117.93 (14)	C24—C23—H23	119.8
C4—C3—P1	120.31 (11)	C22—C23—H23	119.8
C8—C3—P1	121.72 (12)	C25—C24—C23	120.02 (14)
C3—C4—C5	121.64 (15)	C25—C24—H24	120.0
C3—C4—H4	119.2	C23—C24—H24	120.0
C5—C4—H4	119.2	C24—C25—C26	120.15 (16)
C6—C5—C4	119.81 (17)	C24—C25—H25	119.9
C6—C5—H5	120.1	C26—C25—H25	119.9
C4—C5—H5	120.1	C21—C26—C25	120.45 (15)
C7—C6—C5	119.55 (16)	C21—C26—H26	119.8
C7—C6—H6	120.2	C25—C26—H26	119.8
C5—C6—H6	120.2	O1—C27—Cr1	175.10 (12)
C6—C7—C8	120.63 (16)	O2—C28—Cr1	175.62 (14)
C6—C7—H7	119.7	O3—C29—Cr1	177.97 (14)
C8—C7—H7	119.7	O4—C30—Cr1	174.21 (13)
C7—C8—C3	120.44 (16)	C27—Cr1—C29	90.06 (6)
C7—C8—H8	119.8	C27—Cr1—C30	84.22 (6)
C3—C8—H8	119.8	C29—Cr1—C30	90.67 (6)
C14—C9—C10	118.80 (13)	C27—Cr1—C28	87.73 (7)
C14—C9—P1	120.32 (10)	C29—Cr1—C28	91.71 (7)
C10—C9—P1	120.85 (11)	C30—Cr1—C28	171.61 (6)
C11—C10—C9	120.16 (15)	C27—Cr1—P1	98.33 (4)
C11—C10—H10	119.9	C29—Cr1—P1	171.42 (4)
C9—C10—H10	119.9	C30—Cr1—P1	91.97 (4)
C12—C11—C10	120.47 (15)	C28—Cr1—P1	86.83 (5)
C12—C11—H11	119.8	C27—Cr1—P2	177.47 (5)
C10—C11—H11	119.8	C29—Cr1—P2	91.42 (4)
C11—C12—C13	119.81 (14)	C30—Cr1—P2	93.70 (4)
C11—C12—H12	120.1	C28—Cr1—P2	94.28 (5)
C13—C12—H12	120.1	P1—Cr1—P2	80.270 (13)
C12—C13—C14	120.08 (15)	C3—P1—C9	103.03 (6)
C12—C13—H13	120.0	C3—P1—Si1	109.81 (5)
C14—C13—H13	120.0	C9—P1—Si1	106.13 (5)
C13—C14—C9	120.63 (14)	C3—P1—Cr1	120.78 (5)
C13—C14—H14	119.7	C9—P1—Cr1	120.41 (4)
C9—C14—H14	119.7	Si1—P1—Cr1	95.386 (16)
C16—C15—C20	118.32 (14)	C15—P2—C21	102.83 (6)
C16—C15—P2	123.72 (12)	C15—P2—Si1	112.95 (5)
C20—C15—P2	117.92 (11)	C21—P2—Si1	107.65 (5)
C15—C16—C17	120.34 (17)	C15—P2—Cr1	117.58 (5)
C15—C16—H16	119.8	C21—P2—Cr1	120.72 (5)
C17—C16—H16	119.8	Si1—P2—Cr1	94.919 (16)
C18—C17—C16	120.68 (17)	C2—Si1—C1	110.61 (8)
C18—C17—H17	119.7	C2—Si1—P1	110.83 (6)
C16—C17—H17	119.7	C1—Si1—P1	117.22 (6)
C19—C18—C17	119.50 (16)	C2—Si1—P2	106.93 (6)
C19—C18—H18	120.3	C1—Si1—P2	123.42 (6)
C17—C18—H18	120.3	P1—Si1—P2	85.312 (17)
C18—C19—C20	120.47 (17)		
